# Mesoscopic Characterization of the Early Stage of the Glucono-*δ*-Lactone-Induced Gelation of Milk via Image Analysis Techniques

**DOI:** 10.3390/gels9030202

**Published:** 2023-03-06

**Authors:** Kento Sekiguchi, Morimasa Tanimoto, Shuji Fujii

**Affiliations:** 1 Department of Food and Life Sciences, Toyo University, 1-1-1, Izumino, Itakura, Oratown 374-0193, Gunma, Japan; 2 Faculty of Health and Nutrition, Department of Food Sciences, Tokyo Seiei College, 1-4-6, Shinkoiwa, Katsushika, Tokyo 124-8530, Japan

**Keywords:** milk, glucono-δ-lactone, gelation, rheology, microscopy, image analysis

## Abstract

We provide a method for quantifying the kinetics of gelation in milk acidified with glucono-δ-lactone (GDL) using image analysis techniques, particle image velocimetry (PIV), differential variance analysis (DVA) and differential dynamic microscopy (DDM). The gelation of the milk acidified with GDL occurs through the aggregation and subsequent coagulation of the casein micelles as the pH approaches the isoelectric point of the caseins. The gelation of the acidified milk with GDL is an important step in the production of fermented dairy products. PIV qualitatively monitors the average mobility of fat globules during gelation. The gel point estimated by PIV is in good agreement with that obtained by rheological measurement. DVA and DDM methods reveal the relaxation behavior of fat globules during gelation. These two methods make it possible to calculate microscopic viscosity. We also extracted the mean square displacement (MSD) of the fat globules, without following their movement, using the DDM method. The MSD of fat globules shifts to sub-diffusive behavior as gelation progresses. The fat globules used as probes show the change in matrix viscoelasticity caused by the gelling of the casein micelles. Image analysis and rheology can be used complementarily to study the mesoscale dynamics of the milk gel.

## 1. Introduction

Quantifying the gelation of food proteins is important to achieve the desired texture of various food products. The gelation of proteins has been extensively studied using a variety of physical methods such as transmitted electron microscopy (TEM), small-angle X-ray scattering (SAXS), nuclear magnetic resonance (NMR), dynamic light scattering (DLS) and rheological measurements [1,2,3,4,5,6,7,8,9,10,11,12,13,14,15]. Among them, dynamic light scattering (DLS) and rheology are the well-known and most used techniques to characterise gelation behavior. DLS provides the average information about soft-matter dynamics and is widely used to characterize the thermodynamic properties of different systems. Diffusing wave spectroscopy (DWS), known as a derivative of DLS developed for turbid systems, has also been used to characterize the gelation dynamics of casein micelles [11,12,13,14,15,16]. These optical techniques make it possible to determine the gel point and analyze the structure of gel networks [17,18], thus providing information on the temporal evolution of the characteristic length scale of the gel network, such as the mesh size.

Rheological experiments, on the other hand, directly measure various mechanical properties of the systems and are most commonly used to study gelation behavior. Shear-modulus measurements identify the gel point and characterize the stiffness of the gel network. The elasticity of materials is generally given by a simple relationship, $G∼k_{B}T/\xi^{3}$, where ξ is a characteristic length scale, $k_{B}$ is the Boltzmann constant and *T* is the temperature. Since the characteristic mesh size ξ of the casein gel network, roughly a few μm, contributes to the modulus of the milk gel [19,20], the combination of optical microscopy and mechanical methods is essential to understanding how the evolution of the mesoscale structure leads to mechanical properties. However, the application of imaging microscopy to the study of gelation dynamics is still limited. In our recent studies on the rennet-induced gelation of milk, the gelation kinetics was studied using image analysis techniques such as particle image velocimetry (PIV) and differential variance analysis (DVA) by tracking the fat globules as a probe [21,22]. Previous studies have shown that the Brownian motion of fat globules reveals the change in matrix viscoelasticity as a result of the gelation of the casein micelles. The fat globules can be used as a probe to detect the viscoelastic properties of the matrix as a result of gelation. These studies proved that imaging microscopy is a powerful tool to study the mesoscale dynamics of the milk gel.

In this study, we apply the image analysis technique to the gelation of milk acidified with glucono-δ-lactone (GDL). Hydrolysis of GDL to gluconic acid lowers the pH of the milk. When the pH approaches the isoelectric point of the caseins, the casein micelles are destabilized and form the gel network structure. During acidification, the casein micelles undergo dramatic changes in structure and stability as a function of pH [13,23]. As the pH decreases from 6 to 5.2, the net charge of the casein micelles progressively decreases and the calcium phosphate (CaP) nanoclusters gradually dissolve out of the micelles. As recently shown by Moitzi et al, the loss of charge causes the micelles to shrink [4]. A further decrease in pH from 5.2 to 5 leads to the aggregation of the casein micelles, because the α-, β-, and κ-caseins that electrostatically stabilize the micelles collapse completely. These experiments provided us with convincing evidence that the gelation of acidified milk proceeds hierarchically from micro- to macroscopic events. Although it is well-known that acid-induced gelation proceeds hierarchically, the mesoscopic feature linking the micro- and macroscopic behavior of gelation is still unknown. Bacterial fermentation of dairy products such as fresh cheese and yogurt is mimicked by GDL-induced acid gel. To improve the texture and physical propertie of acidified milk products, the mesoscopic information is required. In this article, we show how image analysis techniques can be applied to the gelation of acidified milk. The study of mesoscale dynamics can provide useful information for the quality control of dairy products.

## 2. Results and Discussion

### 2.1. Bulk Rheology

Dynamic viscoelasticity combined with pH measurement provides fundamental information on the gelation of acidified milk. The critical time for gelation was obtained from these data. The time evolution of the shear moduli shows that the gelation of acidified milk can be divided into three steps: incubation time, gelation of the casein micelles and the aging of the acidified milk gel.

**Figure 1:**
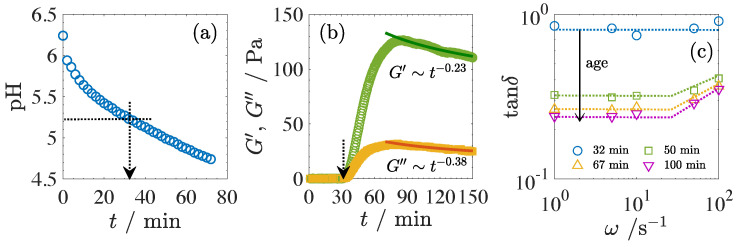
Time evolution of pH value (**a**), time evolution of the storage and loss moduli measured at ω = 1 s${}^{-1}$ (**b**), and tanδ as a function of ω (c). Vertical arrows in panels (**a**,**b**) indicate the time when pH value reached pH = 5.2, a measure of the initiation of the gelation. Solid curves in panel (**b**) correspond to power law relations, $G^{\prime }∼t^{-0.23}$ and $G^{\prime \prime }∼t^{-0.38}$. Dotted lines in panel (**c**) are the guide for eyes. Each symbol corresponds to the data measured at different times, *t*.

Figure 1 shows the time evolution of pH (a), the time evolution of storage shear modulus $G^{\prime }$ and loss modulus $G^{\prime \prime }$ measured at an angular frequency of ω = 1 s${}^{-1}$ (b), and tanδ as a function of angular frequency ω at different elapsed times *t* (c). Here, the elapsed time *t* is the time since GDL was added. The pH value decreases monotonically with *t* due to acidification. It took *t* = 34 min to reach a pH of 5.2, which is a measure of the onset of gelation, i.e., the gel point of the acidified milk [23]. As the gel point is approached, both the shear moduli $G^{\prime }$ and $G^{\prime \prime }$ (Figure 1b) increase abruptly at around *t* = 32 min, which is in reasonable agreement with the time dependence of pH. The appearance of elasticity is apparently due to the percolation of the casein networks. The time evolution of the dynamic shear moduli was also measured at different angular frequencies. All the data obtained at different ω showed the same behavior. In Figure 1c, we plotted tanδ at each elapsed time as a function of the angular frequency used to measure the time evolution of the shear moduli. As shown in Figure 1c, tanδ (= $G^{\prime \prime }$/$G^{\prime }$) is close to 1 at *t* = 32 min and remains constant. This behavior in tanδ is observed in chemically cross-linked gel [24]. Thus, the gel point in this system coincides with the time at which pH = 5.2. This characteristic time, $t_{c}$ = 32 min, could be called the critical time for gelation. Beyond the critical time, $G^{\prime }$ grows more than a hundredfold and tanδ shows a frequency dependence in the high-ω region, as shown in Figure 1a,c.

In the time evolution of the shear moduli in Figure 1b, both moduli reach a local maximum and then gradually decrease according to a power law of time, i.e., $G^{\prime }$∼$t^{-0.23}$ and $G^{\prime \prime }$∼$t^{-0.38}$, respectively. Thus, tanδ decreases with time as tanδ∼$t^{-0.15}$. In addition, tanδ is independent of the gel strength and allows us to focus on the temporal evolution of the casein network. This characteristic has never been observed for the rennet-induced gelation of milk [6,21,22]. The gradual decrease in the shear moduli has been attributed to several causes, such as the redissolution of aggregates due to overacidification [25] and rearrangement of the casein network structure due to the dissociation and reincorporation of caseins based on the dual binding model of caseins [26,27].

The aggregation of casein micelles is induced by the loss of their negative net charge as the pH decreases to the isoelectric point of caseins (pH ∼ 4.6). In the over-acidification, the casein micelles acquire a positive net charge, causing a partial redissolution of the casein aggregates beyond the isoelectric point. In the dual binding model, casein micelles are formed by two couplings: the hydrophobic interactions of casein and intermolecular cross-linking by colloidal calcium phosphate (CaP) nanoclusters, which dissolve around pH = 5.2. The loss of CaP could increase the dissociation of the network structure and, thus, cause the decrease in the modulus. The continued decrease in protein charge with pH allows the internal bonds to reform and rearrange. As the redissolution and rearrangement of casein micelles affects the network structure of caseins and is, thus, related to the aging of the gel network, such as syneresis, the detailed dynamics should be studied in the near future. Rheological measurements show that the gelation of acidified milk can be divided into three steps: the incubation period, and the network formation of the casein micelles and their aging. In the following, we focus only on the early process of gelation, the incubation period and network formation, in acidified milk, but not on the aging dynamics.

### 2.2. Image Analysis

#### 2.2.1. Particle Image Velocimetry (PIV)

PIV is a useful method to visualize the average mobility of the fat globules in the milk [21]. The use of this method provides us with a qualitative understanding of the kinetics, such as the critical time for gelation. The critical time estimated by PIV is in good agreement with $t_{c}$, obtained by rheometry. The displacement fields of the fat globules during gelation were determined using the PIV plugin in ImageJ [28].

**Figure 2:**
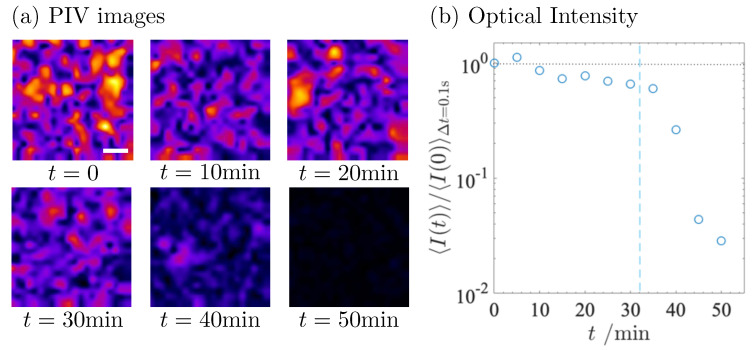
(**a**) PIV images obtained at fixed time lag Δt = 0.1 s at various times *t*. Scale bar corresponds to 10 μm. (**b**) Normalized optical intensity <I(t)>/<I(0)>Δt=0.1 s calculated from PIV image at time lag Δt = 0.1 s as a function of *t*. Vertical dashed line indicates *t* = 32 min.

The fat globules in the milk show vigorous jittering. Following the same procedure as in our previous analysis [21], we analyzed the time evolution of the mobility of the fat globules after the addition of GDL. In Figure 2a, the displacement of the fat globules during the time lag Δt = 0.1 s is shown as a heat map, where the magnitude of the displacement is expressed by color. The brightness of the heat map at *t* = 0 indicates the high activity of the fat globules. It becomes darker as the time *t* elapses after the addition of GDL. The darkness at *t* = 50 min shows that the diffusion of the fat globules is frozen due to the formation of the casein network.

To evaluate the average mobility of the fat globules, the averaged optical intensity over the region of interest (ROI) was calculated using the same procedure as in the previous paper [21]. We also calculated an average of *I*(*t*, δt), and ${\langle I{\left(t\right)}_{\delta t}\rangle }_{t}$, over an ensemble of differential images at different times *t*. Figure 2b shows the time evolution of the averaged optical intensity ${\langle I{\left(t\right)}_{\Delta t=0.1s}\rangle }_{t}$ normalized to that at *t* = 0 min. Here, the time lag, Δt, was fixed at 0.1 s. The normalized optical intensity $\langle I\left(t\right)\rangle /{\langle I\left(0\right)\rangle }_{\Delta t=0.1s}$ in Figure 2b decays suddenly at *t* = 35 min, whereas it decays gradually in the pre-gel state. This characteristic time *t* = 35 min agrees well with the critical time $t_{c}$ = 32 min in the rheometry.

In rennet-induced gelation, PIV analysis clarified the emergence of a mesoscale vacancy in which the serum is confined [21]. The vacancy has a diameter of about 5–10 μm. The formation of the vacancy has also been reported in GDL-induced gel [23,29]. However, we did not find the formation of such a vacancy, even during and after gelation in Figure 2a. Microstructural investigations revealed that the gel made from unheated milk has larger protein clusters, while a highly branched fine structure is formed in the heated milk [23,29]. Lucey et al. proposed that the aggregation of denatured whey proteins during the acidification of heated milk cross-links the casein networks and, thus, affects the microstructure of the casein gel network [29]. Although the experimental conditions in this study did not induce vacancy formation, vacancy formation may be controlled by the physical properties of the casein gel network induced by heat treatment.

PIV shows the qualitative behavior of gelation. The gel point estimated from PIV is in good agreement with the rheology. The optical intensity gradually decreases during the incubation period. PIV detects the decrease in the mobility of the fat globules over the incubation period, indicating the increase in viscosity. Although PIV is useful for detecting qualitative behavior, one would like to have more quantitative information, such as viscosity. In the following sections, we present another technique to extract another aspect of the gelation dynamics.

#### 2.2.2. Differential Variance Analysis (DVA)

DVA provides quantitative information on the relaxation dynamics of the fat globules and the microscopic viscosity of the system, in addition to the critical time for gelation. The relaxation time of the fat globules τ is obtained from an analysis of the relaxation curve obtained by DVA, and the microscopic viscosity of the milk is estimated from the relaxation time even during the incubation period.

**Figure 3:**
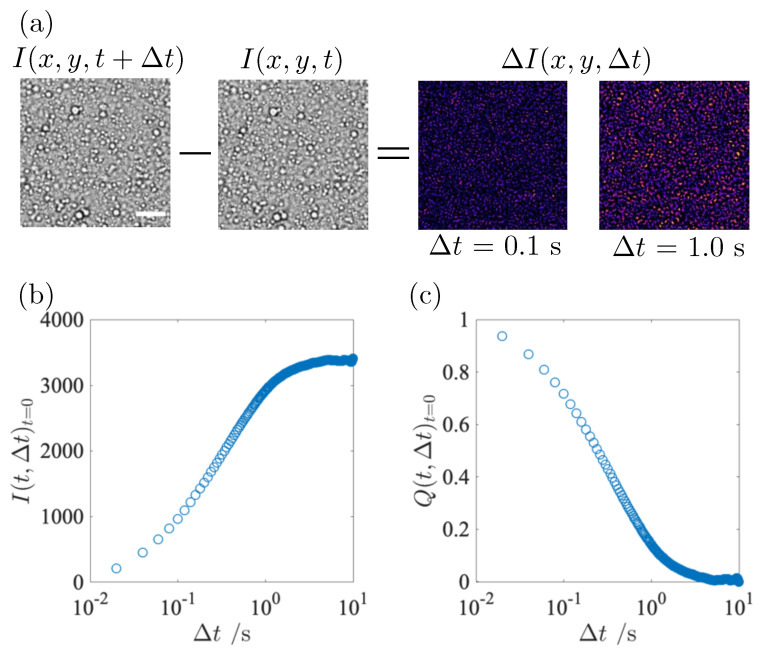
Representative result obtained from DVA analysis. Two images on the left side in (**a**) were taken at interval of time-lag Δt = 0.1 s. Image on the right side shows a differential image at two time lags Δt = 0.1 s and 1.0 s. Scale bar corresponds to 10 μm. As the fat globules move with the interval Δt, bright spots in the differential image becomes large and increase its intensity. Time evolution of the optical intensity I(t,Δt) at *t* = 0 is shown in (**b**). Time evolution of I(t,Δt) is converted to Q(t,Δt) following Equation (1) in (**c**).

The idea of DVA is to isolate only the contribution of moving objects in the sample solution by taking the differences between images separated by a variable time-lag Δt [22,30]. Figure 3a shows two images separated by a time lag Δt and their different images at two time lags Δt = 0.1 and 1.0 s. In the different images, traces of the migration of the fat globules can be seen as spots during Δt. As Δt increases, the spots become clear. It is obvious from the different images that the displacement of the fat globules develops with Δt.

The time evolution of the displacement of the globules allows us to calculate the relation time of the globules. From the definition of the relaxation time τ of colloidal particles, the globules will move a distance comparable to their size on this time scale τ [31]. At time scales longer than τ, the fat globules will move away from their original position and the number of trajectories will increase. The temporal evolution of the different images was estimated quantitatively by the variance of ΔI over the whole image. A representative optical intensity I(t,Δt) at *t* = 0 is shown in Figure 3b. At *t* = 0, the fat globules are freely diffusing. $I{\left(t,\Delta t\right)}_{t=0}$ gradually increases and reaches the plateau value $I_{t,\infty }$ at a sufficiently long Δt compared to the relaxation time. The DVA curve, I(t,Δt), is closely related to the relaxation process of the fat globules and can be used to make the relaxation behavior more intuitive and easier to understand.

We extracted the relaxation time of fat globules from a series of gelation process in the following. Since the relaxation of fat globules is influenced by the casein network formation associated with their gelation, we refer to it as structural relaxation. The following relationship to calculate the DVA dynamic order parameter, Q(t,Δt), is introduced to properly describe the relaxation of the fat globules,

(1) $$Q\left(t,\Delta t\right)=1-\frac{{\langle I\left(t,\Delta t\right)\rangle }_{t}}{I(t,\Delta t_{\infty })}$$

The dynamic order parameter of the DVA at *t* = 0, $Q{\left(t,\Delta t\right)}_{t=0}$, is plotted as a function of Δt in Figure 3c. The single exponential decay in Q(t,Δt) indicates that the DVA, using the fat globules as a probe, captures well the characteristic feature of the averaged mobility of the globules, i.e., the relaxation process. Q(t,Δt) can, therefore, be used to quantitatively describe the structural relaxation of milk.

**Figure 4:**
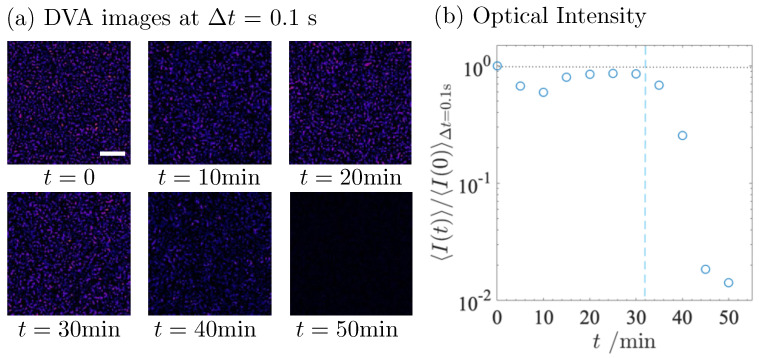
(**a**) DVA images obtained at fixed time-lag Δt = 0.1 s at various times *t*. Scale bar corresponds to 10 μm. (**b**) Normalized optical intensity $\langle I\left(t\right)\rangle /{\langle I\left(0\right)\rangle }_{\Delta t=0.1s}$ calculated from DVA images at time lag Δt = 0.1 s as a function of *t*. Vertical dashed line indicates $t_{c}$ = 32 min.

DVA images with a fixed time lag Δt = 0.1 s at different times *t* are summarised as a sequence with increasing *t* in Figure 4a, and the optical intensity in the DVA images is plotted as a function of *t* in Figure 4b. In the DVA images, it can be seen that the trajectories spread homogeneously in the ROI, which proves that all the fat globules diffuse homogeneously. As *t* = 40 min, it is obvious that the number of trajectories decreases and, finally, the trajectory disappears at *t* = 50 min. The variation in the trajectories is expressed quantitatively by the optical intensity in Figure 4b. The optical intensity normalized over the initial value at *t* = 0 is plotted as a function of *t*. Similar to the PIV data in Figure 2b, the optical intensity decreases abruptly at *t* = 35 min.

**Figure 5:**
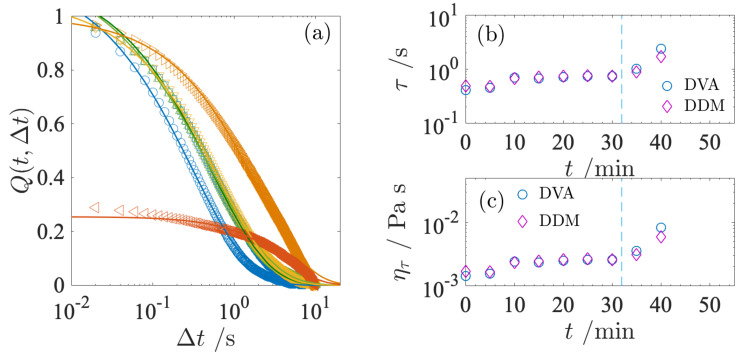
(**a**) DVA curves Q(t,Δt) calculated at different *t*. Solid curves are the best fit to Equation (2). In this panel, each symbol corresponds to 0 min (◯), 10 min (□), 20 min (▵), 30 min (▿), 40 min (▹), and 50 min (◃), respectively. Relaxation time τ obtained from the fitted results is shown in (**b**). Microscopic viscosity $\eta_{\tau }$ calculated is shown in (**c**). In the panel (**b**,**c**), both data obtained from DVA and DDM are plotted. Vertical dashed line shows $t_{c}$ = 32 min.

To assess how the relaxation dynamics of the milk evolve with *t*, the structure relaxation Q(t,Δt) at each *t* is analyzed in Figure 5. In Figure 5a, the Q(t,Δt) curves are plotted at several *t*. The entire curve was fitted to a stretched exponential function,

(2) $$Q\left(t,\Delta t\right)=\exp[-{\left(\Delta t/\tau \right)}^{\beta }],$$

where τ is the relaxation time and β is a stretched exponent. The relaxation curves are well-fitted by Equation (2) with τ and β as two fitting parameters, as can be seen in Figure 5a. The fitting was performed using the numerical computing software Matlab, with the least-square approximation method. The relaxation time τ obtained from the fitting corresponds to a characteristic time for the diffusion of the fat globules. The temporal evolution of Q(t,Δt) characterized by Equation (2) allows evaluation of the relaxation behavior in the gelation process. The τ obtained by fitting the DVA is shown in Figure 5b. DVA curves at *t* = 40 and 50 min do not asymptotically decay smoothly to 0. This is due to the artifact resulting from the insufficient ensemble averaging of the DVA data at long time lags. To resolve this artifact, we need more long-term observations.

During the incubation period, τ increases slightly within 10 min after the addition of GDL and remains almost constant, but increases with *t* beyond $t_{c}$ = 32 min. The diffusion time τ of a spherical particle in a solvent is given by the relationship $\tau =6\pi \eta R^{3}/k_{B}T$ with the viscosity of the suspending medium η, the radius of the particle *R*. Here, we refer to the viscosity estimated from the diffusion time as the microscopic viscosity $\eta_{\tau }$. The fundamental idea of microscopic viscosity is the same as passive microrheology [32]. In passive microrheology, the trajectories of a micron-sized particle embedded in the system as a probe are measured by microscopy. By analyzing the trajectory of diffusing particles, we can calculate the viscoelasticity using the generalized Stokes–Einstein relation. Since the viscoelastic properties of the medium affect the diffusion of the particles, the microscopic viscosity calculated in Figure 5c provides information about the dynamics at the scale of the probe particle. The slowing down of τ can be translated into an increase in microscopic viscosity. It should be noted that the microscopic viscosity at *t* = 0 agrees well with the milk viscosity [22,33]. The DVA method can be useful to monitor the early process of gelation.

DVA is sensitive to the mechanical balance between the strength of the casein network and the Brownian force. A closer look at the time evolution of the microscopic viscosity in the short time region (*t* = 0–10 min) shows that $\eta_{\tau }$ increases slightly at the very beginning of acidification, before $t_{c}$. In the short time region, the pH drops sharply from 6.4 to 5.6. The slight increase in $\eta_{\tau }$ suggests that the rapid decrease in pH induced the local aggregation of the casein micelles. The normalized optical intensity in Figure 4b also decreases slightly 10 min after the addition of GDL, as do the PIV data in Figure 2b. The slight decrease corresponds to an increase in microscopic viscosity. Thereafter, the normalized optical intensity in DVA recovers while the relaxation time remains almost constant. The subsequent recovery behavior is an issue to be clarified.

The stability of casein is highly pH-dependent. Upon acidification, the casein micelles first dissociate into their constituents [4]. The CaP dissociates because lowering the pH increases its aqueous solubility [1]. As the colloidal CaP is responsible for integrating the casein micelles, its dissociation eventually causes the dissociation of the casein molecules. At the same time as the casein micelles dissociate, the casein loses its negative charge. The loss of negative charge leads to intramicellar flocculation, followed by the gelation of the caseins. The ongoing flocculation during acidification can be seen as an increase in microscopic viscosity. The slight increase in $\eta_{\tau }$ is, thus, caused by several factors: the internal changes in the micelles, the loss of CaP and the change in net charge during the pH change.

The DVA method provides us with averaged information on relaxation and access to microscopic viscosity. DVA opens the door to quantifying the gelation dynamics using image analysis techniques. The acquisition of the microrheological property is an advantage of the DVA method. It characterizes the system by diffusion time τ and microscopic viscosity $\eta_{\tau }$ based on real space imaging. Although DVA potentially contains information about the diffusion of particles, it has difficulty in deriving statistical information such as the mean square displacement (MSD) of diffusing particles because DVA tracks the optical intensity but not the motion of the diffusing particle itself. To extract information about the diffusion of the fat globules, we used another effective method which also provides averaged information about the relaxation of the structure in Fourier space.

#### 2.2.3. Differential Dynamic Microscopy (DDM)

Differential dynamic microscopy (DDM) provides us with the relaxation dynamics of fat globules in the same way as DVA. The main difference with DVA is that DDM includes information in wave number space. We extracted the mean square displacement (MSD) information of the fat globules by performing the analysis at the wave number corresponding to their size.

DDM allows us to perform light-scattering experiments using a standard optical microscope and extract quantitative information about the structure and dynamics [34,35]. The basic principle of DDM is similar to that of DVA. DDM works by subtracting two images taken at different time lags Δt in the movie, just as DVA does in Equation (3). The dynamics of the system is obtained from the image structure function D(q,Δt), which is calculated by performing a two-dimensional fast Fourier transform (FFT) analysis on the different images and taking the average over *t* as

(3) $$D\left(q,\Delta t\right)={\langle |FFT\left(\Delta I\left(x,y,t,\Delta t\right)\right)|}^{2}\rangle_{t},$$

where FFT is the FFT operation and *q* is the wavenumber vector. One can, therefore, obtain the Fourier power spectrum of the different images, from which one can obtain the image structure function D(q,Δt) for each wave vector *q* as a function of the time lag Δt. The time evolution of the image structure function is described by

(4) D(q,Δt)=A(q)[1−f(q,Δt)]+B(q).

where f(q,Δt) is the normalized intermediate scattering function, known as the autocorrelation function, which can be measured by DLS experiments. Thus, f(q,Δt) characterizes the relaxation behavior of the system. A(q) is the function related to the static scattering properties of the sample, which also depend on the details of the imaging optics used in this study, and B(q) is a decorrelated background noise and can be regarded as a constant *B*. A time series of the Fourier spectrum of the different images allows the calculation of f(q,Δt) as

(5) $$f\left(q,\Delta t\right)=1-\frac{D(q,\Delta t)-B}{A(q)},$$

DDM can be used as an alternative method for DLS and DWS experiments. In the case of the Brownian particles suspended in a viscoelastic medium, the normalized intermediate scattering function is expected to be,

(6) $$f\left(q,\Delta t\right)=\exp[-{\left(D_{t}q^{2}\Delta t\right)}^{\beta }].$$

Here, $D_{t}$ is the translational diffusion coefficient of the Brownian particles. The average motion of the Brownian particles is represented by their mean square displacement (MSD), $\langle r^{2}\left(\Delta t\right)\rangle$ = $\langle x^{2}\left(\Delta t\right)+y^{2}\left(\Delta t\right)\rangle$, and the distribution of the displacement follows the Gaussian. MSD follows the well-known diffusion behavior

(7) $$\langle r^{2}\left(\Delta t\right)\rangle =4D_{t}\Delta t,$$

Finally, by combining Equations (6) and (7), we obtain a relationship for the MSD of the diffusing particle as follows,

(8) $$\langle r^{2}\left(\Delta t\right)\rangle =\frac{4}{q^{2}}{\left(-\log f\left(q,\Delta t\right)\right)}^{1/\beta },$$

Once we obtain the normalized scattering function from the DDM analysis, it can be translated to MSD. The DDM analysis thus provides us with information on particle diffusion, which is very sensitive to the viscoelastic properties of the surrounding medium.

**Figure 6:**
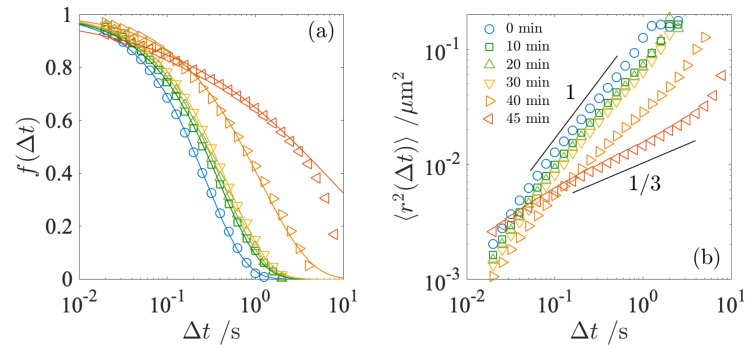
Intermediate scattering function f(Δt) measured at different *t* (**a**). Symbols correspond to the data obtained at different *t*, 0 min (◯), 10 min (□), 20 min (▵), 30 min (▿), 40 min (▹), and 45 min (◃). Solid curves are the best fit to Equation (2). Mean square displacement (MSD) at each *t*, $\langle r^{2}\left(\Delta t\right)\rangle$, is shown in (**b**). In the panel (**b**), two lines with slope of 1 and 1/3 are shown as the guide for eyes.

The normalized intermediate scattering function f(Δt) measured at different *t* is shown in Figure 6. In Figure 6, f(Δt) is plotted at *q* = 10.95 μm${}^{-1}$, corresponding to the averaged radius of the fat globules, so that both results from DVA and DDM can be compared. Here, f(Δt) was fitted with the same equation as in Equation (2) and the relaxation time τ was estimated. The estimated relaxation time was used to calculate the microscopic viscosity $\eta_{\tau }$. The τ and $\eta_{\tau }$ obtained from DDM are compared with the DVA data in Figure 5b,c. We find that these estimates are in good agreement. As mentioned in the previous section, the DVA technique necessitates more long-term observations as the relaxation time slows down. Therefore, we show the microscopic viscosity up to *t* = 40 min and the data at *t* = 45 and 50 min are omitted. Although it is likely that the DDM curve at *t* = 40 min may also include the artifact arising from the insufficient ensemble average at long time lags, the curve in Figure 6a shows a smooth decay, indicating that the measurements at *t* = 40 min remain reliable.

To characterize the diffusive behavior of the fat globules, we calculated the mean square displacement (MSD) of f(Δt), as shown in Figure 6b. As can be seen from Equation (8), the exact form of the intermediate scattering function f(Δt) contains information about the motion on the length scale of the fat globules. The MSD at *t* = 0 increases proportionally with the time delay Δt. The linear relationship, $\langle r^{2}\left(\Delta t\right)\rangle$∼Δt, at *t* = 0–30 min indicates that the fat globules are diffusing in the Newtonian liquid-like environment, i.e., the fat globules exhibit normal diffusion. In the pre-gel state up to *t* = 30 min, the magnitude of the MSD decreases slightly with *t* while the slope remains unity. This is expected, due to the increase in microscopic viscosity, as shown in Figure 5c. The increase in viscosity due to the obstacles created by the local aggregation of the casein micelles reduces the mobility of the fat globules. As gelation progresses with *t*, the slope deviates from unity. After the gelation point, at *t* = 40 min, the slope becomes smaller, which is a signature of the appearance of subdiffusion behavior due to hydrodynamic interactions with the casein network. The diffusion of the fat globules is restricted due to the elasticity of the casein network, resulting in a non-Brownian behavior with a slope less than unity. We see that at *t* = 45 min, the slope decreases to less than unity. Similarly to DVA, due to the extended relaxation time, it was not possible to obtain a sufficient ensemble average at a large time lag Δt, thus resulting in an artifact in the determination of A(q) in Equation (5). However, even taking this into account, there is no doubt that subdiffusion occurred simultaneously with the extended relaxation time, because the slope of the MSD mainly depends on the image structure function D(q,Δt). The diffusion of the fat globules is significantly suppressed and they become more confined once the casein network is firmly developed. The subdiffusive behavior at intermediate Δt indicates that the fat globules are more difficult to move because they are locally trapped within these casein networks. The slower relaxation time of the fat globules results in the higher microscopic viscosity $\eta_{\tau }$. The diffusion properties of the fat globules are significantly influenced by the formation of the casein network.

#### 2.2.4. Application of the Image Analysis

Comparing the image analysis method used in this study with light scattering data presented by another group [36,37], we found that the image analysis data agreed well with each other. The light scattering method detected the growth in casein aggregates at the gel point where the elastic modulus begins to develop. This is consistent with the remarkable decrease in the mobility of fat globules at the gel point obtained by image analysis techniques. We, therefore, expect that image analysis methods will allow us to analyze the gelation behavior in real space, complementing light scattering which gives us information on the dynamics at different length scales.

The present results demonstrate, for the first time, the possibility of measuring the mesoscale dynamics of milk gelation using generic optical microscopy. The effect of polydispersity in the network mesh size and the interaction between casein micelles and fat-globule membranes need to be considered as important factors in the future. The visualization of the casein network itself by confocal microscopy and the application of the particle tracking technique will provide further new insights into understanding the gelling dynamics of acidified milk. The hopping event of the fat globules from the trap could also be directly visualized using the particle tracking technique. There is a critical size of the casein network mesh beyond which the mobility of the fat globules decreases dramatically. As the gelation progresses, hopping becomes more difficult with increasing network confinement. The combination of the direct visualization of the casein network with confocal microscopy and fat-globule dynamics, as obtained in this study, is a future topic. The advances in this study provided by the image analysis techniques will open the door to deeper investigations of the gelling process of food proteins.

## 3. Conclusions

Image analysis techniques provide a unique perspective on the formation of milk gels. We studied the gelation kinetics of acidified milk using several image analysis techniques, particle image velocimetry (PIV), differential variance analysis (DVA) and differential dynamic microscopy (DDM), using fat globules as probes. The gel point estimated from PIV is in good agreement with the rheological data. The relaxation dynamics of the fat globules provided by DVA and DDM allow us to calculate the microscopic viscosity, which is also found to be in good agreement with the bulk viscosity. These techniques revealed that in the very early process of acidification, the microscopic viscosity develops in two steps: The first step occurs immediately after the addition of GDL, which could be attributed to the local aggregation of the casein micelles even in the pre-gel state. In the second step, the microscopic viscosity increases, largely due to the formation of the casein network. These data provide evidence that image analysis is sensitive to mesoscale dynamics during milk gelation.

## 4. Materials and Methods

### 4.1. Sample Preparation

Pasteurized milk at a low temperature, 66 °C for 30 min Takanashi Milk Co. Ltd., Kanagawa, Japan, was purchased from a local supermarket. The mean radius of the fat globules was previously estimated to be <R>=0.57±0.027 μm [21]. Glucono-δ-lactone (GDL) was supplied by FUSO Chemical Co. Ltd, Japan. GDL was added to pasteurized milk at a concentration of 3 wt.%. The mixture was immediately vortexed for 60 s and transferred to a microscope slide or loaded into the rheometer. The solution on the slide was quickly sealed with a coverslip for imaging. The pH value of the mixture was measured using a pH meter, PH71 Yokogawa. In the following, all experiments were carried out at *T* = 25 °C.

### 4.2. Rheometry

For the dynamic shear modulus measurements, a coaxial double cylinder rheometer ONRH-1, OhnaTech Inc, was used. The advantage of this rheometer is that the outer cylinder is made of transparent glass, which allows us to observe the system visually during the measurement. In this study, the time evolution of the shear moduli, the storage modulus $G^{\prime }$ and the loss modulus $G^{\prime \prime }$ were measured at angular frequencies of ω = 1, 5, 10, 50 and 100 s${}^{-1}$ and strain amplitude of γ = 0.03. From each time evolution of $G^{\prime }$ and $G^{\prime \prime }$, we obtained the loss tangent, the ratio of loss to storage modulus, given by tanδ = $G^{\prime \prime }$/$G^{\prime }$, where δ is the phase angle. It is well-known that tanδ follows the time evolution of viscoelasticity in the gelling system [24]. The loss tangent, tanδ, allows us to identify the gel point when it becomes independent of ω around tanδ = 1. In this study, tanδ measured by rheometry was compared with image analysis data.

### 4.3. Light Microscopy

To observe the fat globules during gelation, microscopy was performed using the same combination of equipment as in previous studies [21,22]. In the microscopy, the same position on the slide glass was always observed so that we could correctly evaluate the mobility of the fat globules. All films were recorded at a frame rate of 50 Hz for 10 s. The movies obtained using microscopy were analyzed using different image analysis techniques. We used ImageJ and MATLAB for the image analysis of the obtained movies.

## Data Availability

Not applicable.
